# Supplementary material for: Azithromycin possesses biofilm–inhibitory activity and potentiates non-bactericidal colistin methanesulfonate (CMS) and polymyxin B against Klebsiella pneumonia
Source: PLoS One. 2022 Jul 1;17(7):e0270983. doi: 10.1371/journal.pone.0270983 (PMC9249213; doi:10.1371/journal.pone.0270983)
Supplement: S1 Dataset — This file lists the quantitative replicate data used in the statistical analyses and figures presented in this work. (DOCX) [file pone.0270983.s001.docx]

**Data Sets**

Data sets are listed in the order they appear in the main paper. Missing replicates in data sets are missing data. OD_570_ and A_570_ readings were recorded as three significant figures. Inhibition distances were recorded to the nearest 0.5 mm.

**Data Set 1.**

Test of eight macrolides on the biofilm growth (OD_570_) and metabolic activity (A_570_) of ATCC 10031.

***Macrolide Concentration (mg/L) Replicate Growth (OD_570_) MTT (A_570_)***

Ctrl 0 1 0.446 1.454

Ctrl 0 2 0.485 1.863

Ctrl 0 3 0.446 1.212

ERM 1 1 0.654 1.929

AZM 1 1 0.289 0.786

CLM 1 1 0.551 1.462

ROX 1 1 0.424 1.355

TYL 1 1 0.453 1.321

MID 1 1 0.420 1.373

SPM 1 1 0.373 1.314

JSM 1 1 0.453 1.499

ERM 1 2 0.634 1.971

AZM 1 2 0.284 0.935

CLM 1 2 0.498 1.052

ROX 1 2 0.414 1.399

TYL 1 2 0.426 1.109

MID 1 2 0.409 1.837

SPM 1 2 0.503 1.294

JSM 1 2 0.405 1.402

ERM 1 3 0.585 1.725

AZM 1 3 0.306 1.082

CLM 1 3 0.563 1.539

ROX 1 3 0.520 2.099

TYL 1 3 0.496 1.606

MID 1 3 0.487 1.962

SPM 1 3 0.480 1.301

JSM 1 3 0.433 1.489

ERM 3 1 0.354 1.546

AZM 3 1 0.158 0.556

CLM 3 1 0.360 1.283

ROX 3 1 0.505 1.360

TYL 3 1 0.454 1.445

MID 3 1 0.416 1.353

SPM 3 1 0.331 1.414

JSM 3 1 0.183 0.842

ERM 3 2 0.358 1.356

AZM 3 2 0.152 0.561

CLM 3 2 0.303 1.050

ROX 3 2 0.499 1.392

TYL 3 2 0.452 1.639

MID 3 2 0.364 1.242

SPM 3 2 0.307 1.572

JSM 3 2 0.178 0.886

ERM 3 3 0.345 1.292

AZM 3 3 0.213 0.899

CLM 3 3 0.371 0.840

ROX 3 3 0.418 0.991

TYL 3 3 0.456 1.599

MID 3 3 0.363 1.457

SPM 3 3 0.335 1.335

JSM 3 3 0.191 1.254

ERM 6 1 0.294 0.960

AZM 6 1 0.108 0.560

CLM 6 1 0.258 0.660

ROX 6 1 0.495 1.124

TYL 6 1 0.353 0.872

MID 6 1 0.265 0.951

SPM 6 1 0.256 0.708

JSM 6 1 0.160 0.822

ERM 6 2 0.273 1.157

AZM 6 2 0.109 0.420

CLM 6 2 0.257 0.745

ROX 6 2 0.434 1.233

TYL 6 2 0.350 1.260

MID 6 2 0.278 0.740

SPM 6 2 0.247 1.206

JSM 6 2 0.148 0.847

ERM 6 3 0.287 1.094

AZM 6 3 0.125 0.344

CLM 6 3 0.258 0.627

ROX 6 3 0.478 1.178

TYL 6 3 0.367 1.448

MID 6 3 0.282 0.873

SPM 6 3 0.248 0.740

JSM 6 3 0.149 0.894

ERM 9 1 0.210 1.247

AZM 9 1 0.121 0.499

CLM 9 1 0.212 0.845

ROX 9 1 0.435 1.688

TYL 9 1 0.382 1.182

MID 9 1 0.259 1.162

SPM 9 1 0.239 0.784

JSM 9 1 0.115 0.665

ERM 9 2 0.200 1.135

AZM 9 2 0.126 0.507

CLM 9 2 0.202 0.807

ROX 9 2 0.402 1.546

TYL 9 2 0.316 1.153

MID 9 2 0.266 1.039

SPM 9 2 0.236 0.881

JSM 9 2 0.104 0.680

ERM 9 3 0.296 1.119

AZM 9 3 0.102 0.529

CLM 9 3 0.198 0.736

ROX 9 3 0.390 1.076

TYL 9 3 0.340 0.865

MID 9 3 0.226 0.671

SPM 9 3 0.256 0.609

JSM 9 3 0.101 0.526

**Data Set 2.**

Test of AZM and CMS on the planktonic growth (OD_570_) of ATCC 10031.

***Treatment AZM (mg/L) CMS (mg/L) Replicate Growth (OD_570_)***

AZM 0 / CMS 0 0 0 1 1.145

AZM 0 / CMS 2 0 2 1 1.141

AZM 0 / CMS 4 0 4 1 1.135

AZM 0 / CMS 6 0 6 1 1.128

AZM 0 / CMS 8 0 8 1 1.111

AZM 0 / CMS 12 0 12 1 1.129

AZM 0 / CMS 16 0 16 1 1.123

AZM 0 / CMS 20 0 20 1 1.079

AZM 0 / CMS 24 0 24 1 1.074

AZM 0 / CMS 28 0 28 1 0.970

AZM 0 / CMS 0 0 0 2 1.111

AZM 0 / CMS 2 0 2 2 1.131

AZM 0 / CMS 4 0 4 2 1.124

AZM 0 / CMS 6 0 6 2 1.132

AZM 0 / CMS 8 0 8 2 1.100

AZM 0 / CMS 12 0 12 2 1.137

AZM 0 / CMS 16 0 16 2 1.083

AZM 0 / CMS 20 0 20 2 1.059

AZM 0 / CMS 24 0 24 2 1.055

AZM 0 / CMS 28 0 28 2 1.072

AZM 0 / CMS 0 0 0 3 1.117

AZM 0 / CMS 2 0 2 3 1.120

AZM 0 / CMS 4 0 4 3 1.103

AZM 0 / CMS 6 0 6 3 1.118

AZM 0 / CMS 8 0 8 3 1.117

AZM 0 / CMS 12 0 12 3 1.125

AZM 0 / CMS 16 0 16 3 1.111

AZM 0 / CMS 20 0 20 3 1.069

AZM 0 / CMS 24 0 24 3 1.035

AZM 0 / CMS 28 0 28 3 1.068

AZM 3 / CMS 0 3 0 1 0.418

AZM 3 / CMS 2 3 2 1 0.475

AZM 3 / CMS 4 3 4 1 0.420

AZM 3 / CMS 6 3 6 1 0.158

AZM 3 / CMS 8 3 8 1 0.090

AZM 3 / CMS 12 3 12 1 0.075

AZM 3 / CMS 16 3 16 1 0.101

AZM 3 / CMS 20 3 20 1 0.074

AZM 3 / CMS 24 3 24 1 0.074

AZM 3 / CMS 28 3 28 1 0.074

AZM 3 / CMS 0 3 0 2 0.458

AZM 3 / CMS 2 3 2 2 0.484

AZM 3 / CMS 4 3 4 2 0.417

AZM 3 / CMS 6 3 6 2 0.141

AZM 3 / CMS 8 3 8 2 0.079

AZM 3 / CMS 12 3 12 2 0.079

AZM 3 / CMS 16 3 16 2 0.103

AZM 3 / CMS 20 3 20 2 0.073

AZM 3 / CMS 24 3 24 2 0.077

AZM 3 / CMS 28 3 28 2 0.075

AZM 3 / CMS 0 3 0 3 0.432

AZM 3 / CMS 2 3 2 3 0.526

AZM 3 / CMS 4 3 4 3 0.384

AZM 3 / CMS 6 3 6 3 0.142

AZM 3 / CMS 8 3 8 3 0.092

AZM 3 / CMS 12 3 12 3 0.085

AZM 3 / CMS 16 3 16 3 0.095

AZM 3 / CMS 20 3 20 3 0.073

AZM 3 / CMS 24 3 24 3 0.078

AZM 3 / CMS 28 3 28 3 0.073

AZM 9 / CMS 0 9 0 1 0.276

AZM 9 / CMS 2 9 2 1 0.338

AZM 9 / CMS 4 9 4 1 0.085

AZM 9 / CMS 6 9 6 1 0.082

AZM 9 / CMS 8 9 8 1 0.079

AZM 9 / CMS 12 9 12 1 0.076

AZM 9 / CMS 16 9 16 1 0.079

AZM 9 / CMS 20 9 20 1 0.080

AZM 9 / CMS 24 9 24 1 0.078

AZM 9 / CMS 28 9 28 1 0.075

AZM 9 / CMS 0 9 0 2 0.249

AZM 9 / CMS 2 9 2 2 0.128

AZM 9 / CMS 4 9 4 2 0.088

AZM 9 / CMS 6 9 6 2 0.081

AZM 9 / CMS 8 9 8 2 0.078

AZM 9 / CMS 12 9 12 2 0.076

AZM 9 / CMS 16 9 16 2 0.078

AZM 9 / CMS 20 9 20 2 0.076

AZM 9 / CMS 24 9 24 2 0.079

AZM 9 / CMS 28 9 28 2 0.079

AZM 9 / CMS 0 9 0 3 0.110

AZM 9 / CMS 2 9 2 3 0.181

AZM 9 / CMS 4 9 4 3 0.085

AZM 9 / CMS 6 9 6 3 0.083

AZM 9 / CMS 8 9 8 3 0.078

AZM 9 / CMS 12 9 12 3 0.073

AZM 9 / CMS 16 9 16 3 0.079

AZM 9 / CMS 20 9 20 3 0.075

AZM 9 / CMS 24 9 24 3 0.074

AZM 9 / CMS 28 9 28 3 0.073

**Data Set 3.**

Test of AZM and CMS on the biofilm growth (OD_570_) of ATCC 10031.

***Treatment AZM (mg/L) CMS (mg/L) Replicate Growth (OD_570_)***

AZM 0 / CMS 0 0 0 1 0.898

AZM 0 / CMS 0 0 0 2 0.861

AZM 0 / CMS 0 0 0 3 0.844

AZM 0 / CMS 2 0 2 1 0.787

AZM 0 / CMS 2 0 2 2 0.834

AZM 0 / CMS 2 0 2 3 0.760

AZM 0 / CMS 4 0 4 1 0.628

AZM 0 / CMS 4 0 4 2 0.680

AZM 0 / CMS 4 0 4 3 0.648

AZM 0 / CMS 6 0 6 1 0.491

AZM 0 / CMS 6 0 6 2 0.574

AZM 0 / CMS 6 0 6 3 0.508

AZM 0 / CMS 8 0 8 1 0.375

AZM 0 / CMS 8 0 8 2 0.380

AZM 0 / CMS 8 0 8 3 0.387

AZM 0 / CMS 12 0 12 1 0.245

AZM 0 / CMS 12 0 12 2 0.246

AZM 0 / CMS 12 0 12 3 0.236

AZM 0 / CMS 16 0 16 1 0.221

AZM 0 / CMS 16 0 16 2 0.252

AZM 0 / CMS 16 0 16 3 0.232

AZM 0 / CMS 20 0 20 1 0.224

AZM 0 / CMS 20 0 20 2 0.230

AZM 0 / CMS 20 0 20 3 0.232

AZM 0 / CMS 24 0 24 1 0.241

AZM 0 / CMS 24 0 24 2 0.261

AZM 0 / CMS 24 0 24 3 0.238

AZM 0 / CMS 28 0 28 1 0.195

AZM 0 / CMS 28 0 28 2 0.195

AZM 0 / CMS 28 0 28 3 0.207

AZM 3 / CMS 0 3 0 1 0.497

AZM 3 / CMS 0 3 0 2 0.466

AZM 3 / CMS 0 3 0 3 0.445

AZM 3 / CMS 2 3 2 1 0.368

AZM 3 / CMS 2 3 2 2 0.337

AZM 3 / CMS 2 3 2 3 0.341

AZM 3 / CMS 4 3 4 1 0.277

AZM 3 / CMS 4 3 4 2 0.296

AZM 3 / CMS 4 3 4 3 0.323

AZM 3 / CMS 6 3 6 1 0.258

AZM 3 / CMS 6 3 6 2 0.271

AZM 3 / CMS 6 3 6 3 0.289

AZM 3 / CMS 8 3 8 1 0.237

AZM 3 / CMS 8 3 8 2 0.236

AZM 3 / CMS 8 3 8 3 0.251

AZM 3 / CMS 12 3 12 1 0.270

AZM 3 / CMS 12 3 12 2 0.182

AZM 3 / CMS 12 3 12 3 0.202

AZM 3 / CMS 16 3 16 1 0.268

AZM 3 / CMS 16 3 16 2 0.290

AZM 3 / CMS 16 3 16 3 0.235

AZM 3 / CMS 20 3 20 1 0.213

AZM 3 / CMS 20 3 20 2 0.194

AZM 3 / CMS 20 3 20 3 0.203

AZM 3 / CMS 24 3 24 1 0.225

AZM 3 / CMS 24 3 24 2 0.217

AZM 3 / CMS 24 3 24 3 0.189

AZM 3 / CMS 28 3 28 1 0.202

AZM 3 / CMS 28 3 28 2 0.239

AZM 3 / CMS 28 3 28 3 0.208

AZM 9 / CMS 0 9 0 1 0.183

AZM 9 / CMS 0 9 0 2 0.192

AZM 9 / CMS 0 9 0 3 0.195

AZM 9 / CMS 2 9 2 1 0.190

AZM 9 / CMS 2 9 2 2 0.201

AZM 9 / CMS 2 9 2 3 0.195

AZM 9 / CMS 4 9 4 1 0.205

AZM 9 / CMS 4 9 4 2 0.208

AZM 9 / CMS 4 9 4 3 0.197

AZM 9 / CMS 6 9 6 1 0.190

AZM 9 / CMS 6 9 6 2 0.193

AZM 9 / CMS 6 9 6 3 0.197

AZM 9 / CMS 8 9 8 1 0.240

AZM 9 / CMS 8 9 8 2 0.198

AZM 9 / CMS 8 9 8 3 0.206

AZM 9 / CMS 12 9 12 1 0.206

AZM 9 / CMS 12 9 12 2 0.20

AZM 9 / CMS 12 9 12 3 0.198

AZM 9 / CMS 16 9 16 1 0.211

AZM 9 / CMS 16 9 16 2 0.205

AZM 9 / CMS 16 9 16 3 0.187

AZM 9 / CMS 20 9 20 1 0.230

AZM 9 / CMS 20 9 20 2 0.227

AZM 9 / CMS 20 9 20 3 0.201

AZM 9 / CMS 24 9 24 1 0.220

AZM 9 / CMS 24 9 24 2 0.203

AZM 9 / CMS 24 9 24 3 0.199

AZM 9 / CMS 28 9 28 1 0.212

AZM 9 / CMS 28 9 28 2 0.209

AZM 9 / CMS 28 9 28 3 0.208

**Data Set 4.**

Test of AZM and CMS on the metabolic activity (A_570_) of ATCC 10031.

***Treatment AZM (mg/L) CMS (mg/L) Replicate MTT (A_570_)***

AZM 0 / CMS 0 0 0 1 1.862

AZM 0 / CMS 0 0 0 2 1.743

AZM 0 / CMS 0 0 0 3 1.484

AZM 0 / CMS 2 0 2 1 1.843

AZM 0 / CMS 2 0 2 2 1.164

AZM 0 / CMS 2 0 2 3 2.012

AZM 0 / CMS 4 0 4 1 1.937

AZM 0 / CMS 4 0 4 2 1.171

AZM 0 / CMS 4 0 4 3 1.525

AZM 0 / CMS 6 0 6 1 1.247

AZM 0 / CMS 6 0 6 2 1.134

AZM 0 / CMS 6 0 6 3 1.583

AZM 0 / CMS 8 0 8 1 1.022

AZM 0 / CMS 8 0 8 2 1.575

AZM 0 / CMS 8 0 8 3 1.466

AZM 0 / CMS 12 0 12 1 0.943

AZM 0 / CMS 12 0 12 2 0.768

AZM 0 / CMS 12 0 12 3 0.548

AZM 0 / CMS 16 0 16 1 0.372

AZM 0 / CMS 16 0 16 2 0.263

AZM 0 / CMS 16 0 16 3 0.240

AZM 3 / CMS 0 3 0 1 0.674

AZM 3 / CMS 0 3 0 2 0.911

AZM 3 / CMS 0 3 0 3 0.658

AZM 3 / CMS 2 3 2 1 0.631

AZM 3 / CMS 2 3 2 2 0.539

AZM 3 / CMS 2 3 2 3 0.606

AZM 3 / CMS 4 3 4 1 0.309

AZM 3 / CMS 4 3 4 2 0.345

AZM 3 / CMS 4 3 4 3 0.387

AZM 3 / CMS 6 3 6 1 0.166

AZM 3 / CMS 6 3 6 2 0.177

AZM 3 / CMS 6 3 6 3 0.227

AZM 3 / CMS 8 3 8 1 0.209

AZM 3 / CMS 8 3 8 2 0.234

AZM 3 / CMS 8 3 8 3 0.220

AZM 3 / CMS 12 3 12 1 0.202

AZM 3 / CMS 12 3 12 2 0.159

AZM 3 / CMS 12 3 12 3 0.249

AZM 3 / CMS 16 3 16 1 0.194

AZM 3 / CMS 16 3 16 2 0.159

AZM 3 / CMS 16 3 16 3 0.178

AZM 9 / CMS 0 9 0 1 0.341

AZM 9 / CMS 0 9 0 2 0.297

AZM 9 / CMS 0 9 0 3 0.306

AZM 9 / CMS 2 9 2 1 0.185

AZM 9 / CMS 2 9 2 2 0.217

AZM 9 / CMS 2 9 2 3 0.193

AZM 9 / CMS 4 9 4 1 0.156

AZM 9 / CMS 4 9 4 2 0.159

AZM 9 / CMS 4 9 4 3 0.192

AZM 9 / CMS 6 9 6 1 0.172

AZM 9 / CMS 6 9 6 2 0.136

AZM 9 / CMS 6 9 6 3 0.139

AZM 9 / CMS 8 9 8 1 0.170

AZM 9 / CMS 8 9 8 2 0.182

AZM 9 / CMS 8 9 8 3 0.152

AZM 9 / CMS 12 9 12 1 0.218

AZM 9 / CMS 12 9 12 2 0.153

AZM 9 / CMS 12 9 12 3 0.152

AZM 9 / CMS 16 9 16 1 0.144

AZM 9 / CMS 16 9 16 2 0.183

AZM 9 / CMS 16 9 16 3 0.134

**Data Set 5.**

Test of AZM and CMS on further biofilm growth (change in OD_570_) of ATCC 10031.

***Treatment AZM (mg/L) CMS (mg/L) Replicate Initial OD_570_ Final OD_570_***

AZM 0 / CMS 0 0 0 1 0.749 0.906

AZM 0 / CMS 0 0 0 2 0.708 0.913

AZM 0 / CMS 0 0 0 3 0.671 0.877

AZM 0 / CMS 8 0 8 1 0.614 0.844

AZM 0 / CMS 8 0 8 2 0.644 0.945

AZM 0 / CMS 8 0 8 3 0.684 0.975

AZM 0 / CMS 16 0 16 1 0.641 0.853

AZM 0 / CMS 16 0 16 2 0.574 0.876

AZM 9 / CMS 0 9 0 2 0.515 0.636

AZM 9 / CMS 0 9 0 3 0.492 0.626

AZM 9 / CMS 8 9 8 1 0.539 0.582

AZM 9 / CMS 8 9 8 2 0.435 0.492

AZM 9 / CMS 16 9 16 1 0.561 0.567

AZM 9 / CMS 16 9 16 2 0.466 0.489

AZM 9 / CMS 16 9 16 3 0.481 0.570

**Data Set 6.**

Test of AZM on the biofilm growth (OD_570_) of UHI strains.

***Strain AZM (mg/L) Replicate Growth (OD_570_)***

UHI 117 0 1 1.092

UHI 117 0 2 1.036

UHI 117 0 3 1.075

UHI 117 9 1 0.764

UHI 117 9 2 0.793

UHI 117 9 3 0.799

UHI 117 18 1 0.806

UHI 117 18 2 0.783

UHI 117 18 3 0.792

UHI 329 0 1 0.904

UHI 329 0 2 0.809

UHI 329 0 3 0.833

UHI 329 9 1 0.713

UHI 329 9 2 0.757

UHI 329 9 3 1.150

UHI 329 18 1 0.694

UHI 329 18 2 0.694

UHI 329 18 3 0.869

UHI 486 0 1 1.125

UHI 486 0 2 1.164

UHI 486 0 3 1.149

UHI 486 9 1 0.978

UHI 486 9 2 0.971

UHI 486 9 3 0.912

UHI 486 18 1 0.895

UHI 486 18 2 0.845

UHI 486 18 3 0.809

UHI 489 0 1 1.115

UHI 489 0 2 1.102

UHI 489 0 3 1.114

UHI 489 9 1 0.818

UHI 489 9 2 0.756

UHI 489 9 3 0.750

UHI 489 18 1 0.791

UHI 489 18 2 0.802

UHI 489 18 3 0.801

UHI 509 0 1 0.964

UHI 509 0 2 0.961

UHI 509 0 3 0.979

UHI 509 9 1 0.807

UHI 509 9 2 0.817

UHI 509 9 3 0.838

UHI 509 18 1 0.757

UHI 509 18 2 0.759

UHI 509 18 3 0.754

UHI 519 0 1 1.223

UHI 519 0 2 1.172

UHI 519 0 3 1.190

UHI 519 9 1 0.882

UHI 519 9 2 0.777

UHI 519 9 3 0.761

UHI 519 18 1 0.763

UHI 519 18 2 0.814

UHI 519 18 3 0.739

UHI 520 0 1 1.139

UHI 520 0 2 1.380

UHI 520 0 3 1.102

UHI 520 9 1 0.834

UHI 520 9 2 0.750

UHI 520 9 3 0.840

UHI 520 18 1 0.671

UHI 520 18 2 0.720

UHI 520 18 3 0.789

UHI 1090 0 1 1.058

UHI 1090 0 2 1.090

UHI 1090 0 3 1.103

UHI 1090 9 1 1.151

UHI 1090 9 2 1.115

UHI 1090 9 3 1.121

UHI 1090 18 1 0.882

UHI 1090 18 2 0.891

UHI 1090 18 3 0.884

UHI 1609 0 1 0.736

UHI 1609 0 2 0.789

UHI 1609 0 3 0.787

UHI 1609 9 1 0.737

UHI 1609 9 2 0.751

UHI 1609 9 3 0.774

UHI 1609 18 1 0.627

UHI 1609 18 2 0.617

UHI 1609 18 3 0.615

UHI 1633 0 1 1.033

UHI 1633 0 2 1.405

UHI 1633 0 3 1.122

UHI 1633 9 1 0.630

UHI 1633 9 2 0.845

UHI 1633 9 3 0.765

UHI 1633 18 1 0.637

UHI 1633 18 2 0.677

UHI 1633 18 3 0.778

UHI 1667 0 1 1.154

UHI 1667 0 2 1.550

UHI 1667 0 3 1.504

UHI 1667 9 1 0.925

UHI 1667 9 2 1.277

UHI 1667 9 3 0.959

UHI 1667 18 1 0.555

UHI 1667 18 2 0.704

UHI 1667 18 3 0.535

**Data Set 7.**

Test of AZM and CMS on biofilm growth (OD_570_) of UHI strains.

***Strain AZM (mg/L) CMS (mg/L) Replicate Growth (OD_570_)***

UHI 117 0 0 1 1.092

UHI 117 0 8 1 0.992

UHI 117 0 12 1 0.866

UHI 117 9 0 1 0.764

UHI 117 9 8 1 0.674

UHI 117 9 12 1 0.638

UHI 117 0 0 2 1.036

UHI 117 0 8 2 0.973

UHI 117 0 12 2 0.819

UHI 117 9 0 2 0.793

UHI 117 9 8 2 0.637

UHI 117 9 12 2 0.685

UHI 117 0 0 3 1.075

UHI 117 0 8 3 0.974

UHI 117 0 12 3 0.835

UHI 117 9 0 3 0.799

UHI 117 9 8 3 0.650

UHI 117 9 12 3 0.591

UHI 329 0 0 1 0.904

UHI 329 0 0 2 0.809

UHI 329 0 0 3 0.833

UHI 329 0 12 1 0.572

UHI 329 0 12 2 0.529

UHI 329 0 12 3 0.531

UHI 329 0 8 1 0.728

UHI 329 0 8 2 0.769

UHI 329 0 8 3 0.613

UHI 329 9 0 1 0.713

UHI 329 9 0 2 0.757

UHI 329 9 0 3 1.150

UHI 329 9 12 1 0.300

UHI 329 9 12 2 0.318

UHI 329 9 12 3 0.323

UHI 329 9 8 1 0.372

UHI 329 9 8 2 0.399

UHI 329 9 8 3 0.364

UHI 486 0 0 1 1.125

UHI 486 0 8 1 1.200

UHI 486 0 12 1 1.104

UHI 486 9 0 1 0.978

UHI 486 9 8 1 0.898

UHI 486 9 12 1 0.884

UHI 486 0 0 2 1.164

UHI 486 0 8 2 1.207

UHI 486 0 12 2 1.061

UHI 486 9 0 2 0.971

UHI 486 9 8 2 0.859

UHI 486 9 12 2 0.743

UHI 486 0 0 3 1.149

UHI 486 0 8 3 1.213

UHI 486 0 12 3 1.080

UHI 486 9 0 3 0.912

UHI 486 9 8 3 0.871

UHI 486 9 12 3 0.794

UHI 489 0 0 1 1.115

UHI 489 0 8 1 1.015

UHI 489 0 12 1 0.836

UHI 489 9 0 1 0.818

UHI 489 9 8 1 0.772

UHI 489 9 12 1 0.595

UHI 489 0 0 2 1.102

UHI 489 0 8 2 0.986

UHI 489 0 12 2 0.811

UHI 489 9 0 2 0.756

UHI 489 9 8 2 0.652

UHI 489 9 12 2 0.604

UHI 489 0 0 3 1.114

UHI 489 0 8 3 0.981

UHI 489 0 12 3 0.805

UHI 489 9 0 3 0.750

UHI 489 9 8 3 0.726

UHI 489 9 12 3 0.568

UHI 509 0 0 1 0.964

UHI 509 0 8 1 0.898

UHI 509 0 12 1 0.872

UHI 509 9 0 1 0.807

UHI 509 9 8 1 0.799

UHI 509 9 12 1 0.681

UHI 509 0 0 2 0.961

UHI 509 0 8 2 0.920

UHI 509 0 12 2 0.851

UHI 509 9 0 2 0.817

UHI 509 9 8 2 0.831

UHI 509 9 12 2 0.678

UHI 509 0 0 3 0.979

UHI 509 0 8 3 0.941

UHI 509 0 12 3 0.745

UHI 509 9 0 3 0.838

UHI 509 9 8 3 0.782

UHI 509 9 12 3 0.692

UHI 519 0 0 1 1.223

UHI 519 0 8 1 1.093

UHI 519 0 12 1 0.851

UHI 519 9 0 1 0.882

UHI 519 9 8 1 0.713

UHI 519 9 12 1 0.638

UHI 519 0 0 2 1.172

UHI 519 0 8 2 1.023

UHI 519 0 12 2 0.759

UHI 519 9 0 2 0.777

UHI 519 9 8 2 0.657

UHI 519 9 12 2 0.619

UHI 519 0 0 3 1.190

UHI 519 0 8 3 1.088

UHI 519 0 12 3 0.767

UHI 519 9 0 3 0.761

UHI 519 9 8 3 0.599

UHI 519 9 12 3 0.673

UHI 520 0 0 1 1.139

UHI 520 0 8 1 0.995

UHI 520 0 12 1 0.716

UHI 520 9 0 1 0.834

UHI 520 9 8 1 0.792

UHI 520 9 12 1 0.706

UHI 520 0 0 2 1.380

UHI 520 0 8 2 0.930

UHI 520 0 12 2 0.796

UHI 520 9 0 2 0.750

UHI 520 9 8 2 0.801

UHI 520 9 12 2 0.707

UHI 520 0 0 3 1.102

UHI 520 0 8 3 0.893

UHI 520 0 12 3 0.731

UHI 520 9 0 3 0.840

UHI 520 9 8 3 0.832

UHI 520 9 12 3 0.676

UHI 1090 0 0 1 1.058

UHI 1090 0 0 2 1.090

UHI 1090 0 0 3 1.103

UHI 1090 0 12 1 1.399

UHI 1090 0 12 2 1.167

UHI 1090 0 12 3 1.324

UHI 1090 0 8 1 1.103

UHI 1090 0 8 2 1.098

UHI 1090 0 8 3 1.106

UHI 1090 9 0 1 1.151

UHI 1090 9 0 2 1.115

UHI 1090 9 0 3 1.121

UHI 1090 9 12 1 0.934

UHI 1090 9 12 2 0.649

UHI 1090 9 12 3 0.854

UHI 1090 9 8 1 0.806

UHI 1090 9 8 2 0.874

UHI 1090 9 8 3 0.795

UHI 1609 0 0 1 0.736

UHI 1609 0 0 2 0.789

UHI 1609 0 0 3 0.787

UHI 1609 0 12 1 0.366

UHI 1609 0 12 2 0.322

UHI 1609 0 12 3 0.325

UHI 1609 0 8 1 0.646

UHI 1609 0 8 2 0.631

UHI 1609 0 8 3 0.645

UHI 1609 9 0 1 0.627

UHI 1609 9 0 2 0.617

UHI 1609 9 0 3 0.615

UHI 1609 9 12 1 0.240

UHI 1609 9 12 2 0.264

UHI 1609 9 12 3 0.217

UHI 1609 9 8 1 0.266

UHI 1609 9 8 2 0.266

UHI 1609 9 8 3 0.222

UHI 1633 0 0 1 1.033

UHI 1633 0 0 2 1.405

UHI 1633 0 0 3 1.122

UHI 1633 0 12 1 0.807

UHI 1633 0 12 2 1.169

UHI 1633 0 12 3 0.800

UHI 1633 0 8 1 0.842

UHI 1633 0 8 2 1.278

UHI 1633 0 8 3 0.879

UHI 1633 9 0 1 0.630

UHI 1633 9 0 2 0.845

UHI 1633 9 0 3 0.765

UHI 1633 9 12 1 0.643

UHI 1633 9 12 2 0.621

UHI 1633 9 12 3 0.588

UHI 1633 9 8 1 0.633

UHI 1633 9 8 2 0.631

UHI 1633 9 8 3 0.627

UHI 1667 0 0 1 1.154

UHI 1667 0 0 2 1.550

UHI 1667 0 0 3 1.504

UHI 1667 0 12 1 0.629

UHI 1667 0 12 2 0.625

UHI 1667 0 12 3 0.540

UHI 1667 0 8 1 0.836

UHI 1667 0 8 2 0.856

UHI 1667 0 8 3 0.803

UHI 1667 9 0 1 0.925

UHI 1667 9 0 2 1.277

UHI 1667 9 0 3 0.959

UHI 1667 9 12 1 0.537

UHI 1667 9 12 2 0.489

UHI 1667 9 12 3 0.451

UHI 1667 9 8 1 0.483

UHI 1667 9 8 2 0.478

UHI 1667 9 8 3 0.528

**Data Set 8.**

Inhibition (mm) of growth of UHI strains around polymyxin B disks on AZM plates.

***Strain AZM (mg/L) Replicate Inhibition (mm)***

UHI 117 0 1 15.0

UHI 117 0 2 14.0

UHI 117 0 3 15.0

UHI 117 3 1 15.0

UHI 117 3 2 16.0

UHI 117 3 3 15.0

UHI 117 6 1 17.0

UHI 117 6 2 17.0

UHI 117 6 3 16.0

UHI 117 9 1 17.0

UHI 117 9 2 18.0

UHI 117 9 3 18.0

UHI 329 0 1 17.0

UHI 329 0 2 16.0

UHI 329 0 3 17.0

UHI 329 3 1 17.0

UHI 329 3 2 18.0

UHI 329 3 3 18.0

UHI 329 6 1 17.0

UHI 329 6 2 18.0

UHI 329 6 3 17.0

UHI 329 9 1 18.0

UHI 329 9 2 18.0

UHI 329 9 3 18.0

UHI 486 0 1 13.0

UHI 486 0 2 13.0

UHI 486 0 3 13.5

UHI 486 3 1 14.0

UHI 486 3 2 15.0

UHI 486 6 1 15.0

UHI 486 6 2 16.0

UHI 486 9 1 17.0

UHI 486 9 2 16.0

UHI 489 0 1 15.5

UHI 489 0 2 15.0

UHI 489 0 3 15.0

UHI 489 3 1 16.0

UHI 489 3 2 16.0

UHI 489 3 3 16.0

UHI 489 6 1 16.0

UHI 489 6 2 17.0

UHI 489 6 3 17.0

UHI 489 9 1 19.0

UHI 489 9 2 19.0

UHI 489 9 3 18.0

UHI 509 0 1 13.5

UHI 509 0 2 14.0

UHI 509 0 3 14.0

UHI 509 3 1 14.0

UHI 509 3 2 14.0

UHI 509 3 3 14.0

UHI 509 6 2 17.5

UHI 509 6 3 17.0

UHI 509 9 1 23.0

UHI 509 9 2 25.0

UHI 509 9 3 25.0

UHI 519 0 1 13.0

UHI 519 0 2 13.5

UHI 519 0 3 13.5

UHI 519 3 1 13.0

UHI 519 3 2 13.0

UHI 519 3 3 13.0

UHI 519 6 1 15.0

UHI 519 6 2 15.0

UHI 519 6 3 15.0

UHI 519 9 1 17.0

UHI 519 9 2 16.0

UHI 519 9 3 16.0

UHI 520 0 1 13.0

UHI 520 0 2 13.0

UHI 520 0 3 13.0

UHI 520 3 1 14.0

UHI 520 3 2 14.5

UHI 520 3 3 14.5

UHI 520 6 1 15.5

UHI 520 6 2 15.0

UHI 520 6 3 15.0

UHI 520 9 1 15.0

UHI 520 9 2 16.0

UHI 520 9 3 16.5

UHI 1090 0 1 17.0

UHI 1090 0 2 20.0

UHI 1090 0 3 17.0

UHI 1090 3 1 16.0

UHI 1090 3 2 16.0

UHI 1090 3 3 16.0

UHI 1090 6 1 17.0

UHI 1090 6 2 18.0

UHI 1090 6 3 16.0

UHI 1090 9 1 19.0

UHI 1090 9 2 17.0

UHI 1090 9 3 18.0

UHI 1609 0 1 15.0

UHI 1609 0 2 15.0

UHI 1609 0 3 15.0

UHI 1609 3 1 18.0

UHI 1609 3 2 18.0

UHI 1609 3 3 18.0

UHI 1609 6 1 18.0

UHI 1609 6 2 18.0

UHI 1609 6 3 18.0

UHI 1609 9 1 19.0

UHI 1609 9 2 19.0

UHI 1609 9 3 18.0

UHI 1633 0 1 0.0

UHI 1633 0 2 0.0

UHI 1633 0 3 0.0

UHI 1633 3 1 0.0

UHI 1633 3 2 0.0

UHI 1633 3 3 0.0

UHI 1633 6 1 0.0

UHI 1633 6 2 0.0

UHI 1633 6 3 0.0

UHI 1633 9 1 0.0

UHI 1633 9 2 0.0

UHI 1633 9 3 0.0

UHI 1667 0 1 17.0

UHI 1667 0 2 16.0

UHI 1667 0 3 17.0

UHI 1667 3 1 18.0

UHI 1667 3 2 18.0

UHI 1667 3 3 19.0

UHI 1667 6 1 18.0

UHI 1667 6 2 18.0

UHI 1667 6 3 18.0

UHI 1667 9 1 19.0

UHI 1667 9 2 18.0

UHI 1667 9 3 18.0
