# Supplementary material for: Azithromycin possesses biofilm–inhibitory activity and potentiates non-bactericidal colistin methanesulfonate (CMS) and polymyxin B against Klebsiella pneumonia
Source: PLoS One. 2022 Jul 1;17(7):e0270983. doi: 10.1371/journal.pone.0270983 (PMC9249213; doi:10.1371/journal.pone.0270983)
Supplement: S1 Fig — Macrolides show different levels of inhibition of K. pneumonia ATCC 10031 biofilm growth and can be visualised by Hierarchical cluster analysis that group strains according to similarity. Dendrograms were constructed using mean relative (A) optical density (OD570) and (B) metabolic activity (A570) measurements of biofilm growth after 24 h. Macrolides linked to the same node (small circles) are more similar than those linked by deeper nodes, and the arbitrary root for this dendrogram is indicated by the dashed circle. (PPTX) [file pone.0270983.s002.pptx]

## Slide 1
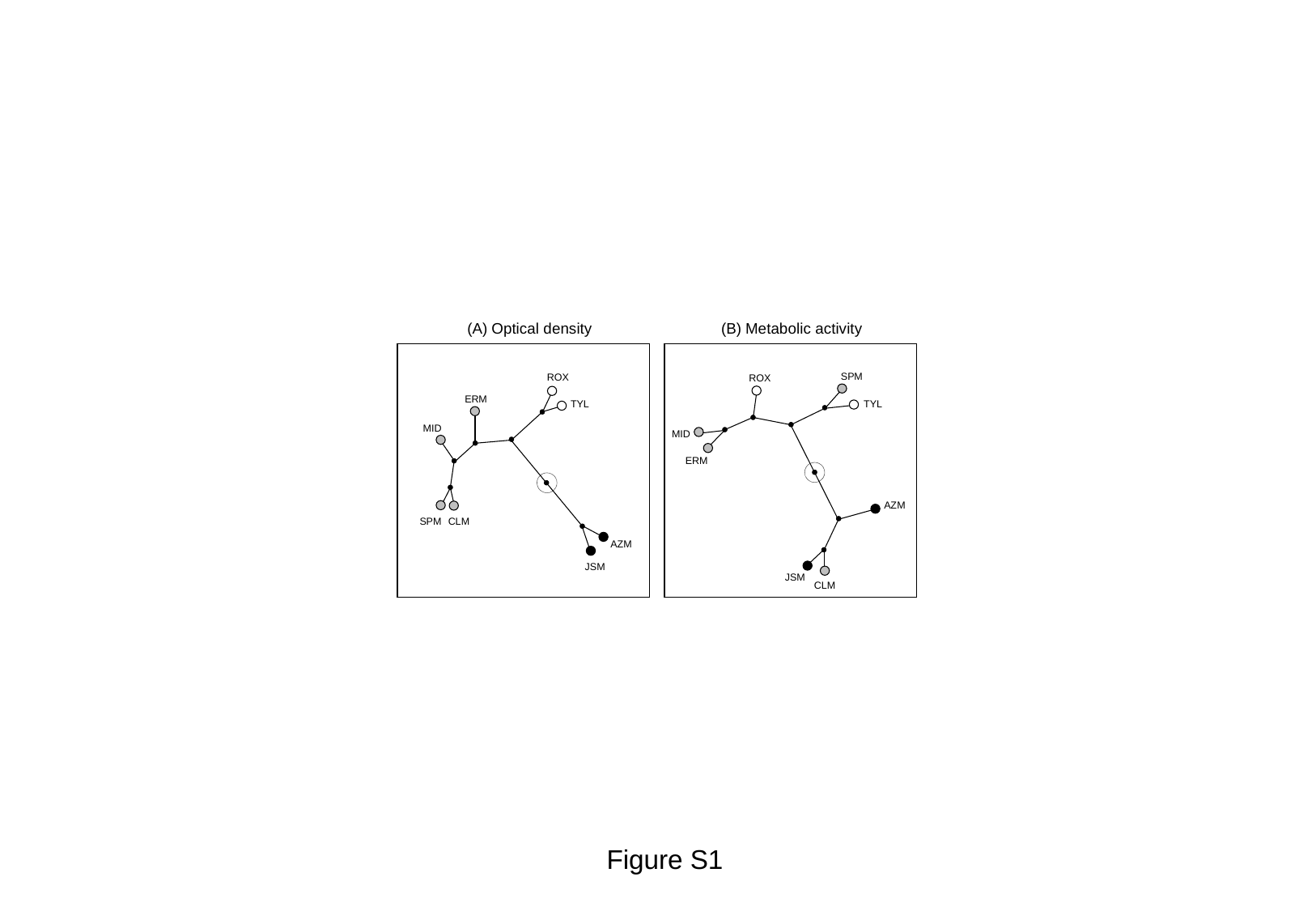

(A) Optical density
(B) Metabolic activity
SPM
ROX
ROX
ERM
TYL
TYL
MID
MID
ERM
AZM
SPM
CLM
AZM
JSM
JSM
CLM
Figure S1
