# Supplementary figures and images for: Azithromycin possesses biofilm–inhibitory activity and potentiates non-bactericidal colistin methanesulfonate (CMS) and polymyxin B against Klebsiella pneumonia
Source: PLoS One. 2022 Jul 1;17(7):e0270983. doi: 10.1371/journal.pone.0270983 (PMC9249213; doi:10.1371/journal.pone.0270983)

## Slide 1
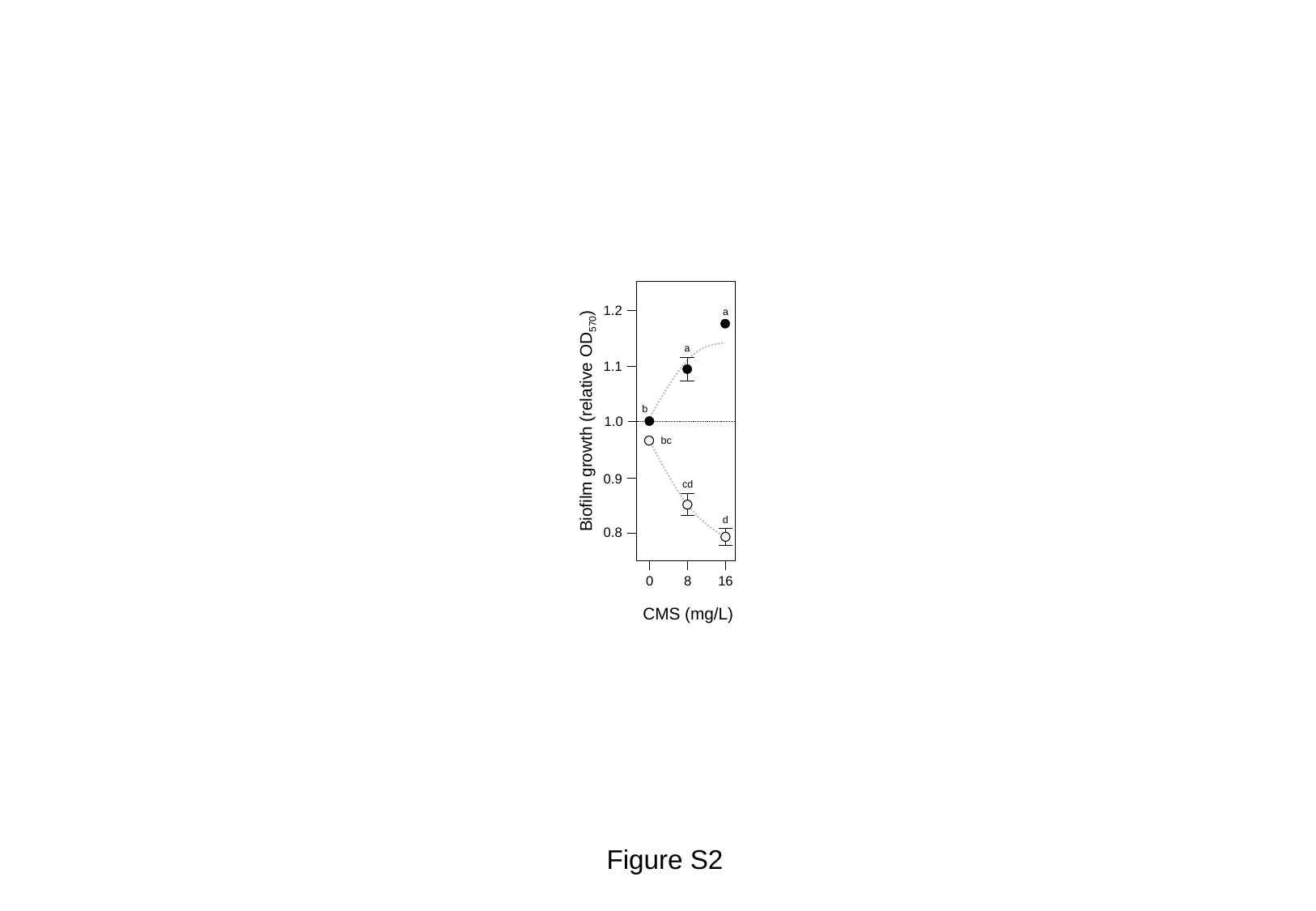

1.2
a
a
1.1
b
Biofilm growth (relative OD570)
1.0
bc
0.9
cd
d
0.8
0
8
16
CMS (mg/L)
Figure S2

Supplement: S2 Fig — The effect of azithromycin (AZM) and colistin methanesulfonate (CSM) on established K. pneumonia ATCC 10031 biofilms was investigated by optical density (OD570) measurements after 24 h incubation. Mean relative OD570 ± SE (n = 3) is shown, with no-AZM (control) treatments indicated by dark circles and 9 mg/L AZM treatments by light circles (trends are suggested by dashed curves). Means not connected by the same letters are significantly different (LSMeans Differences Tukey HSD, alpha = 0.05 for mixed-effects model of relative OD570). (PPTX) [file pone.0270983.s003.pptx]

## Slide 1
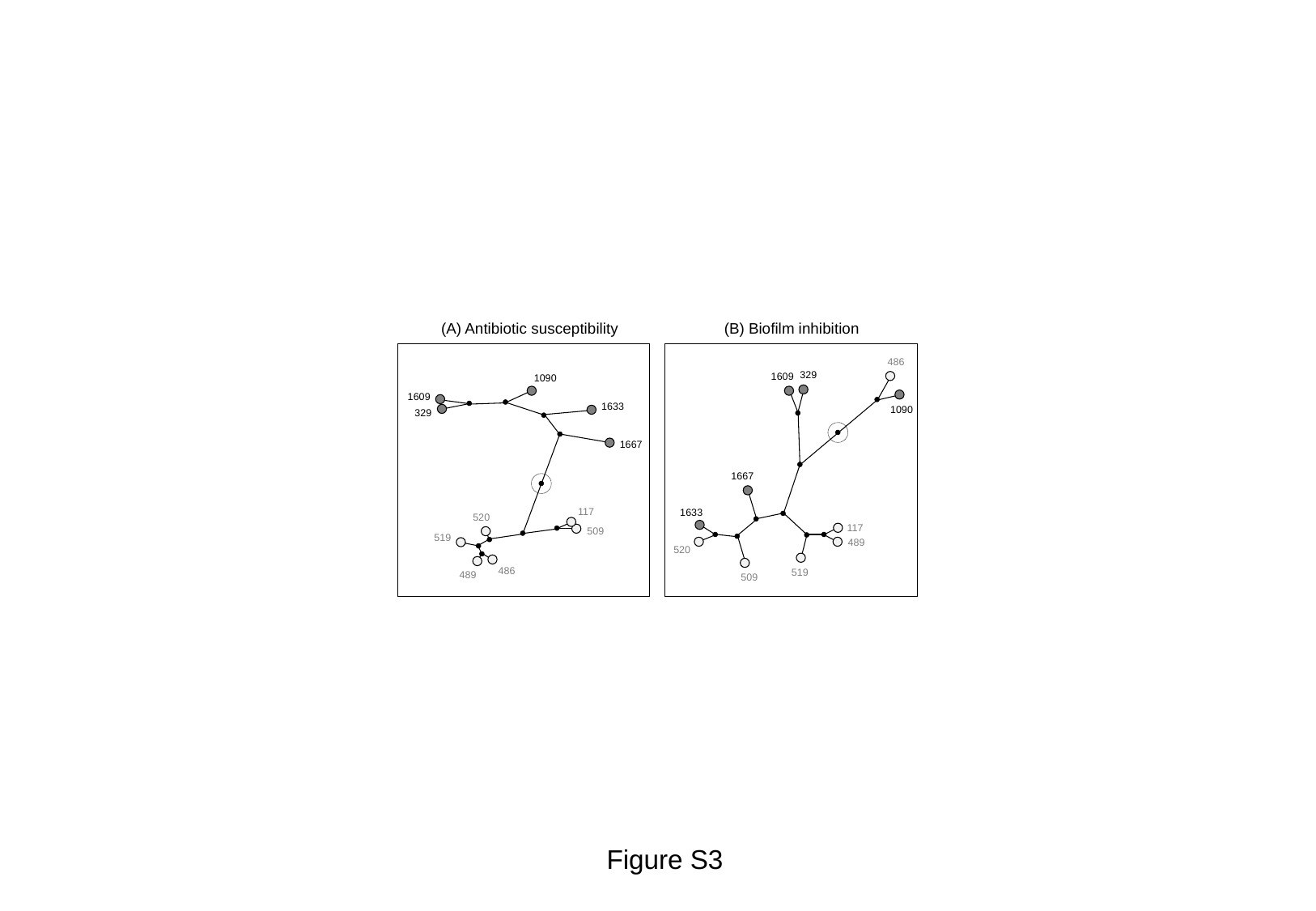

(A) Antibiotic susceptibility
(B) Biofilm inhibition
1090
1609
1633
329
1667
117
520
509
519
486
489
486
329
1609
1090
1667
1633
117
489
520
519
509
Figure S3

Supplement: S3 Fig — The phenotypic diversity among the multiple-drug resistant (MDR) and non-MDR Ukrainian Hospital Isolate (UHI) strains can be visualised by Hierarchical cluster analysis that group strains according to similarity of (A) antibiotic susceptibility and (B) the effect of azithromycin (AZM) on biofilm growth in dendrograms with different topologies. The dendrogram on the left was constructed using disc-diffusion assay data for those antibiotics listed in S1 Table and the one on the right using mean relative optical density (OD570) measurements of biofilm growth with AZM after 24 h. MDR strains are indicated by dark circles and non-MDR strains by light circles. Strains linked to the same node (small circles) are more similar than those linked by deeper nodes, and the arbitrary roots for these dendrograms are indicated by the dashed circle. (PPTX) [file pone.0270983.s004.pptx]
