# Supplementary material for: Azithromycin possesses biofilm–inhibitory activity and potentiates non-bactericidal colistin methanesulfonate (CMS) and polymyxin B against Klebsiella pneumonia
Source: PLoS One. 2022 Jul 1;17(7):e0270983. doi: 10.1371/journal.pone.0270983 (PMC9249213; doi:10.1371/journal.pone.0270983)
Supplement: S4 Fig — The effect of azithromycin (AZM) on biofilm growth of multiple-drug resistant (MDR) and non-MDR K. pneumonia Ukrainian Hospital Isolate (UHI) strains was investigated by biofilm assays with optical density (OD570) measurements after 24 h incubation. Data are shown as mean OD570 ± SE (n = 3) (trends are suggested by dashed curves). Means not connected by the same letters are significantly different (LSMeans Differences Tukey HSD, alpha = 0.05 for individual UHI strain mixed-effects models of OD570; no significant effect for UHI 329, p = 0.06). Strains not connected by the same letters indicated in parentheses in the top-left of each panel are significantly different (LSMeans Differences Tukey HSD, alpha = 0.05 for combined UHIO strains mixed-effects model of relative inhibition). (PPTX) [file pone.0270983.s005.pptx]

## Slide 1
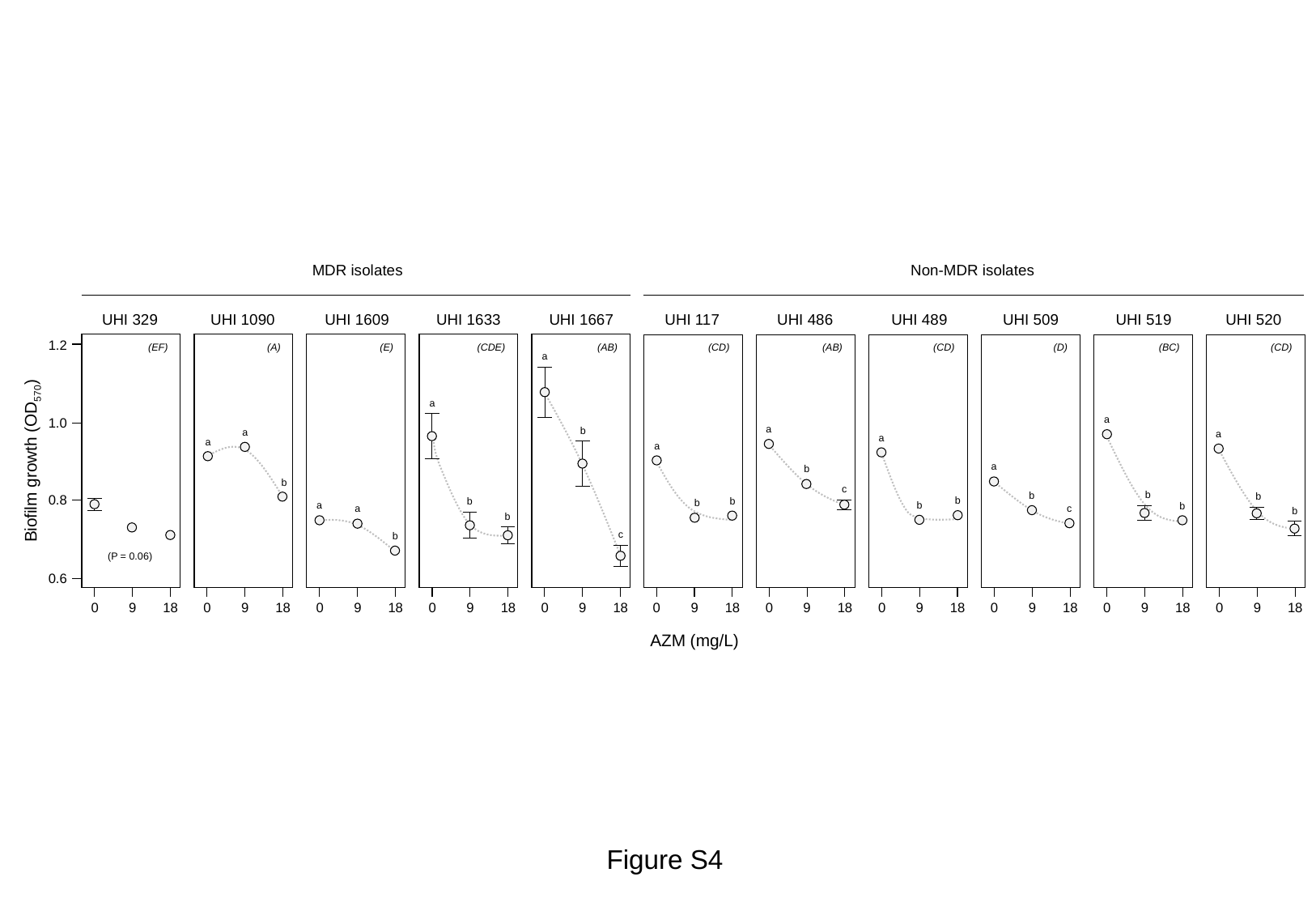

MDR isolates
Non-MDR isolates
UHI 329
UHI 1090
UHI 1609
UHI 1633
UHI 1667
UHI 117
UHI 486
UHI 489
UHI 509
UHI 519
UHI 520
1.2
(EF)
(A)
(E)
(CDE)
(AB)
(CD)
(AB)
(CD)
(D)
(BC)
(CD)
a
a
a
1.0
a
b
a
a
a
a
a
Biofilm growth (OD570)
a
b
b
c
b
b
b
0.8
b
b
b
b
b
a
b
c
a
b
b
c
b
(P = 0.06)
0.6
0
9
18
0
9
18
0
9
18
0
9
18
0
9
18
0
9
18
0
9
18
0
9
18
0
9
18
0
9
18
0
9
18
AZM (mg/L)
Figure S4
