# Supplementary material for: Azithromycin possesses biofilm–inhibitory activity and potentiates non-bactericidal colistin methanesulfonate (CMS) and polymyxin B against Klebsiella pneumonia
Source: PLoS One. 2022 Jul 1;17(7):e0270983. doi: 10.1371/journal.pone.0270983 (PMC9249213; doi:10.1371/journal.pone.0270983)
Supplement: S1 Table — This table lists the origins and antibiotic susceptibility of the UHI strains used in this work. (DOCX) [file pone.0270983.s006.docx]

| **S1 Table.** Origin and antibiotic susceptibility of the UHI *Klebsiella pneumoniae* strains used in this work. | | | | | | | | | | | | |
| --- | --- | --- | --- | --- | --- | --- | --- | --- | --- | --- | --- | --- |
|  |  | *Non-MDR UHI strains* | | | | | | *MDR UHI strains* | | | | |
|  | | **117** | **486** | **489** | **509** | **519** | **520** | **329** | **1090** | **1609** | **1633** | **1667** |
| **Origin**^1^ | Sample^2^ | S | F | F | F | F | F | F | F | P | P | S |
|  | Patient diagnosis^3^ | B | E | E | E | E | E | E | E | O | O | S |
| **Antibiotic sensitivity**^4^ | Amoxicillin^5^ | R | R | R | R | R | R | R | R | R | R | R |
|  | Ampicillin^6^ | S | S | S | S | S | S | R | R | R | R | R |
|  | Amykacin | S | S | S | S | S | S | R | R | R | R | R |
|  | Azithromycin^7^ | 6 | 10 | 10 | 20 | 18 | 18 | 17 | 17 | 15 | 13^8^ | 17 |
|  | Gentamicin | S | S | S | S | S | S | R | R | R | R | R |
|  | Cefepime | S | S | S | S | S | S | R | R | R | R | R |
|  | Cefoperazone-sulbactam | S | S | S | S | S | S | R | R | R | R | R |
|  | Cefotaxime | S | S | S | S | S | S | R | R | R | R | R |
|  | Ceftazidime | S | S | S | S | S | S | R | R | R | R | R |
|  | Ceftriaxone | S | S | S | S | S | S | R | R | R | R | R |
|  | Cefuroxime | R | S | S | R | S | S | R | R | R | R | R |
|  | Chloramphenicol | S | S | S | S | S | S | S | S | S | R | S |
|  | Ciprofloxacin | S | S | S | S | S | S | R | R | R | R | S |
|  | Ertapenem | S | S | S | S | S | S | R | R | R | R | R |
|  | Gatifloxacin | S | S | S | S | S | S | R | R | R | R | S |
|  | Imipenem | S | S | S | S | S | S | R | R | R | R | R |
|  | Levofloxacin | S | S | S | S | S | S | R | R | R | R | S |
|  | Meropenem | S | S | S | S | S | S | R | R | R | R | R |
|  | Ofloxacin | S | S | S | S | S | S | R | R | R | R | S |
|  | Piperacillin-tazobactam | S | S | S | S | S | S | R | R | R | R | R |
|  | Polymyxin B^9^ | S | S | S | S | S | S | S | S | S | R | S |
|  | Tigecycline | S | S | S | S | S | S | R | S | S | I | S |
|  | Tobramycin | S | S | S | S | S | S | R | R | R | R | R |
|  | Trimetoprima^10^ | S | S | S | S | S | S | R | R | R | S | R |
|  | Coliston (broth)^11^ | S | S | S | S | S | S | S | R | S | R | S |
| **Beta-lactamase genes** | NDM | - | + | + | + | + | + | + | - | - | - | + |
|  | VIM | - | - | - | - | - | - | - | - | - | - | - |
|  | KPC | - | + | - | + | - | + | - | - | - | + | + |
|  | OXA-48-like | + | - | - | - | - | - | - | - | - | - | - |
| **Enzyme activity** | Cephalosporinase AmpC |  |  |  |  |  |  | + | + | + | + | - |
|  | Carbapenemase KPC |  |  |  |  |  |  | + | - | + | + | + |
|  | Carbapenemase MBL |  |  |  |  |  |  | + | + | + | - | - |
|  | Carbapenemase OXA-48 |  |  |  |  |  |  | + | + | + | - | - |
|  | ESBL^13^ |  |  |  |  |  |  | + | + | + | + | + |
| ^1^ Non-MDR UHI strains are from Moshynets et al. (2020) and MDR UHI strains are from the Center for Pediatric Cardiology and Cardiac Surgery, Kiev, Ukraine. ^2^ Strains were recovered from sputum (S), faeces (f) and the pharynx (P). ^3^ Patient diagnoses were bronchial asthma (B) or enteritis (E) or samples were from on-receipt inspection (O). ^4^ Antibiotic susceptibilities determined from disc-diffusion assays are shown as are sensitive (S), intermediate (susceptible, increased exposure) (I) or resistant (R). ^5^ Also known as clavulanate; ^6^ Also known as sulbactam; ^7^ Disc-diffusion inhibition diameters (mm) are provided for Azithromycin as there is no official EUCAST break-point for *K. pneumoniae* which is ‘officially’ resistant; in these assays, 6 mm suggests complete resistance while 15 – 20 mm suggests significant sensitivity. ^8^ UHI 1633 growth looked abnormal and rather transparent compared to areas away from the disk. ^9^ Polymyxin B is the official alternative to Colistin/CMS for disc-diffusion testing. ^10^ Also known as sulfametoxazol. ^11^ Colistin broth microdilution assay; sensitive is ≤ 1 mg/L. ^12^ AmpliSens MDR KPOXA-48-FRT / MBL-FTR PCR (+, positive’ -, negative). ^13^ Extended spectrum beta-lactamase (+, positive’ -, negative). No tests were undertaken for those cells left blank. | | | | | | | | | | | | |
